# The co-occurrence of pulmonary embolism and aortic valve thrombosis complicated by myocardial infarction as a clinical manifestation of antithrombin III deficiency: a case report

**DOI:** 10.1093/ehjcr/ytaf332

**Published:** 2025-07-12

**Authors:** Wojciech Kula, Krzysztof Sus, Tomasz Wołyniak, Katarzyna Ciężkowska, Jakub Drozd

**Affiliations:** Cardiology Department, Hospital of the Ministry of the Interior and Administration, ul. Grenadierów 3, Lublin 20-331, Poland; Cardiology Department, Hospital of the Ministry of the Interior and Administration, ul. Grenadierów 3, Lublin 20-331, Poland; Cardiology Department, Hospital of the Ministry of the Interior and Administration, ul. Grenadierów 3, Lublin 20-331, Poland; Cardiology Department, Hospital of the Ministry of the Interior and Administration, ul. Grenadierów 3, Lublin 20-331, Poland; Cardiology Department, Hospital of the Ministry of the Interior and Administration, ul. Grenadierów 3, Lublin 20-331, Poland

**Keywords:** Native aortic valve thrombosis, Type 2 myocardial infarction, Pulmonary embolism, Thrombophilia, Case report

## Abstract

**Background:**

Hypercoagulable states may lead to vascular complications in both the systemic and pulmonary systems, and these conditions may even co-occur. Thrombosis of the native aortic valve, unlike thrombotic events on prosthetic valves, is a very rare condition that may result from thrombophilia and can mimic other valvular disorders. Most significantly, it can serve as a source of further embolic events.

**Case summary:**

A 48-year-old man, a smoker, diagnosed in the emergency department with a small acute pulmonary embolism, was referred to the cardiology department for further treatment. After a transthoracic echocardiogram showed regional contractility dysfunction of the left ventricle, the patient underwent coronary angiography. Following the detection of acute occlusion of the posterior descending branch of the right coronary artery, an effective balloon coronary angioplasty was performed. Due to the suspicion of embolic aetiology of the myocardial infarction, the diagnostic workup was expanded to include a transoesophageal echocardiogram, which revealed a pathological structure on the aortic valve. Ultimately, thrombosis of a normally structured native aortic valve, coexisting with a slight pulmonary embolism, was diagnosed.

**Discussion:**

The low incidence of native aortic valve thrombosis as a manifestation of thrombophilia, along with its transient nature, may lead to the underestimation of this clinical issue and presents challenges in diagnostic and treatment workflows, especially in case of co-occurring pulmonary embolism.

Learning points

- Venous thromboembolic events concomitant with systemic embolic complications do not always indicate paradoxical embolism; they may instead be an expression of a hypercoagulable state.
- Although native aortic valve thrombosis remains a casuistry, it should first be considered as a manifestation of thrombophilia and second as a potential source of remote arterial embolization; the transient nature of native aortic thrombosis may lead to incomplete diagnosis in patients with thromboembolic events in both the pulmonary and systemic circulation.

## Introduction

The coexistence of venous thromboembolism and arterial thrombosis in a single individual is rare in clinical practice. It is usually associated with arteriovenous shunts, such as a patent foramen ovale, atrial septal defect, or pulmonary arteriovenous fistula, which can lead to paradoxical embolism, sometimes presenting as a type 2 myocardial infarction (MI) due to coronary artery embolization.^1^ On the other hand, concurrent thromboembolic complications in both pulmonary and systemic circulation, without a pathological connection between them, may result from thrombophilia, although such occurrences are extremely rare.^2^

## Summary figure

**Figure:**
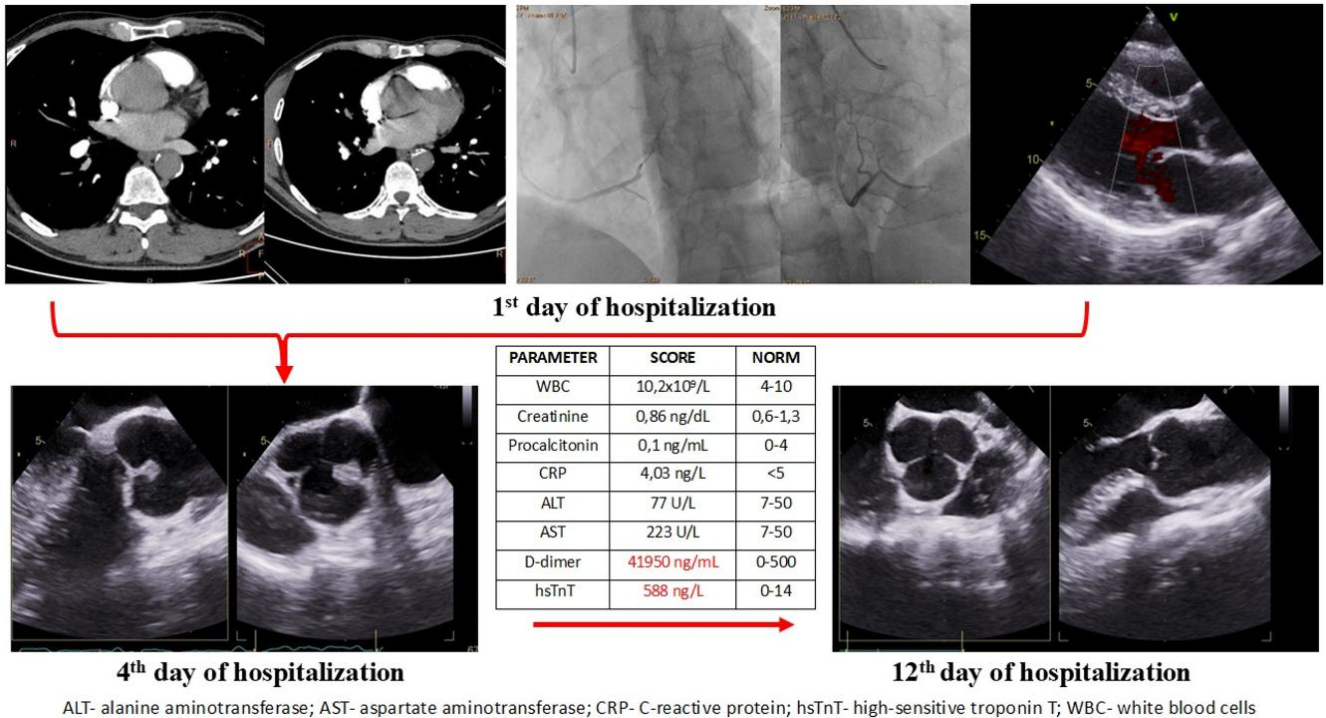
Diagnostic challenges and the transient nature of native aortic valve thrombosis illustrated by consecutively performed tests.

## Case presentation

**Figure 1:**
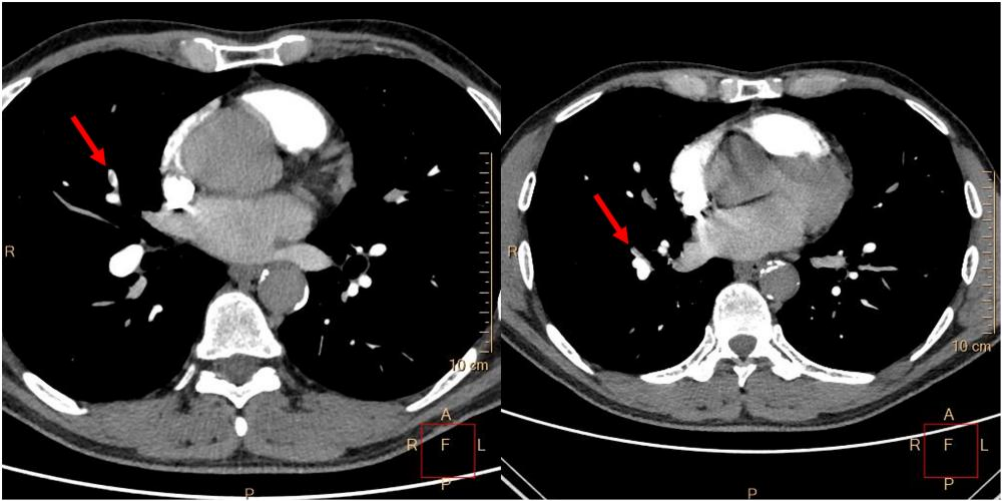
Computed tomography pulmonary angiogram: small thrombi in the fifth and eighth pulmonary segments of the right lung (red arrows).

**Figure 2:**
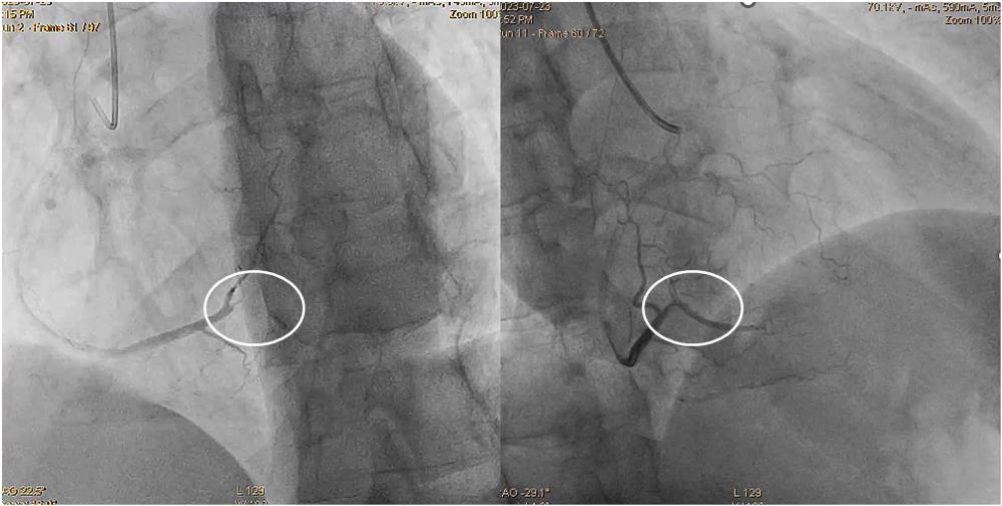
Coronary angiography focused on the posterior descending artery (white circle): posterior descending artery occlusion (left side) and posterior descending artery patency after percutaneous coronary intervention (right side).

**Figure 3:**
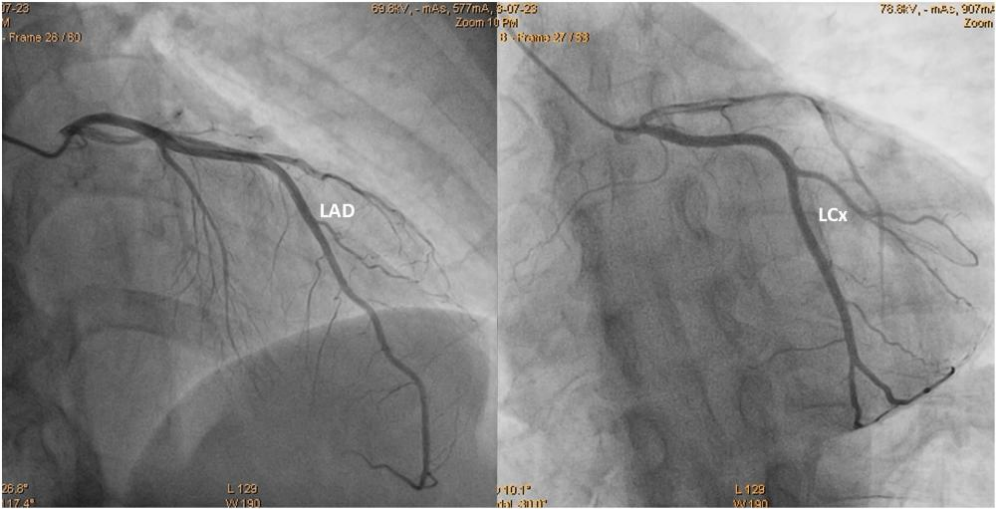
The angiogram of the left coronary artery showing no stenoses.

A 48-year-old male smoker, without a history of chronic diseases, was admitted to the emergency department due to chest pain lasting 4 h. Q waves in the inferior leads and no ST segment abnormalities were observed on the electrocardiogram (ECG). Significantly increased D-dimer (41 950 ng/mL; normal range up to 500) and high-sensitivity troponin T concentrations (588 ng/L; normal range up to 500) triggered the performance of a chest angio-computed tomography (CT). Small thrombi in the fifth and eighth segments of the right lung were confirmed (*Figure 1*), and the patient was referred to the cardiology department. At admission, oxygen saturation (SpO_2_) was 98%, heart rate was 103 b.p.m., and blood pressure was 131/71 mmHg. The Pulmonary Embolism Severity Index (PESI) score was 58 points, indicating a 30-day mortality risk of 0%–1.6%, which corresponds to very low risk. The simplified PESI score of 0 points also classified the patient as low risk for mortality, with a 1.1% risk. Normal right ventricle function, no enlargement of heart chambers, no valvular diseases, and akinesis of basal segments of the inferior wall were found on transthoracic echocardiography. These findings resulted in the performance of an invasive coronary angiography. Acute occlusion of the posterior descending branch of the right coronary artery was treated by thrombectomy and balloon angioplasty. A semi-compliant, non-drug coated balloon with a diameter of 2.25 mm was inflated to a pressure of 12 atm. Given the optimized angiographic results, which demonstrated thrombolysis in myocardial infarction 3 flow without dissection or residual stenosis, and considering the small vessel diameter of less than 2.5 mm, no stent was implanted (*Figure 2*). Aspirin, clopidogrel, a beta-blocker, and a statin were initiated, as well as enoxaparin using treatment dosages. Transoesophageal echocardiography (TEE) was recommended in connection with the normal angiogram of the left coronary artery (*Figure 3*) and suspicion of the thromboembolic nature of the MI.^3^

**Figure 4:**
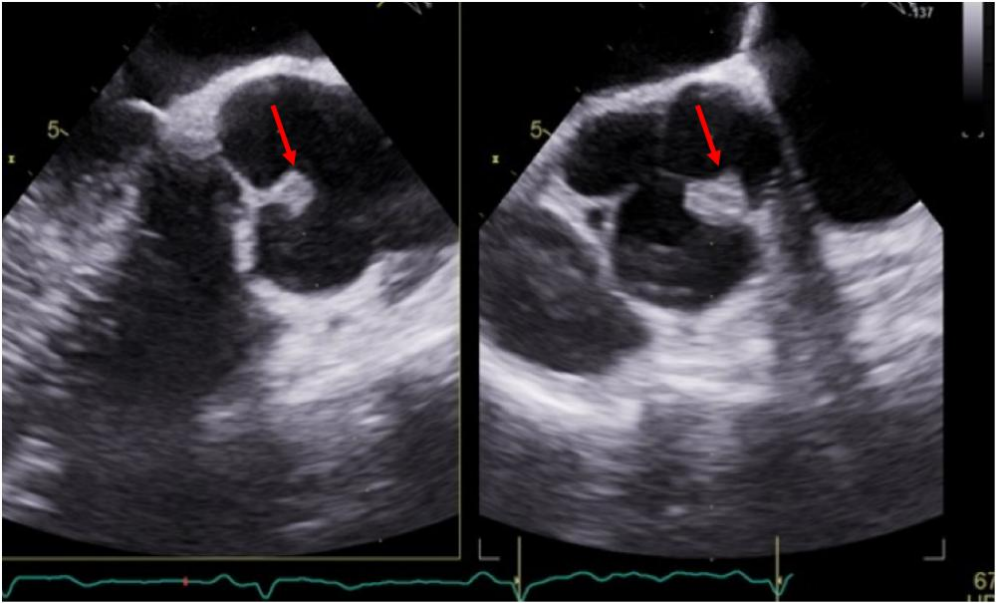
Transoesophageal echocardiography: pathological structure 8 × 12 mm fixed on the right/non-coronary commissure of the native tricuspid aortic valve (red stars).

Transoesophageal echocardiography performed 3 days later excluded thrombi in the left atrium and intra- and extracardiac shunts. Transoesophageal echocardiography was able to confirm an irregular, mobile structure measuring 8 × 12 mm on the right/non-coronary commissure of the tricuspid aortic valve (*Figure 4*). Based on this finding, a differential diagnosis of thrombus, vegetation, and tumour was considered.^4^ Microbial blood cultures were performed prior to the initiation of antibiotics because there were no fever and low levels of inflammatory markers. Ultimately, microbiological blood tests were determined to be negative after 10 days of culture. Also, no arrhythmic events of atrial fibrillation/fluttering were observed during the few days of monitoring, and negative screening tests for connective tissue disorders were observed. On an ultrasound test of the deep venous lower limbs and pelvis, no abnormalities were found, as well as no embolic consequences on CT of the central nervous system.

**Figure 5:**
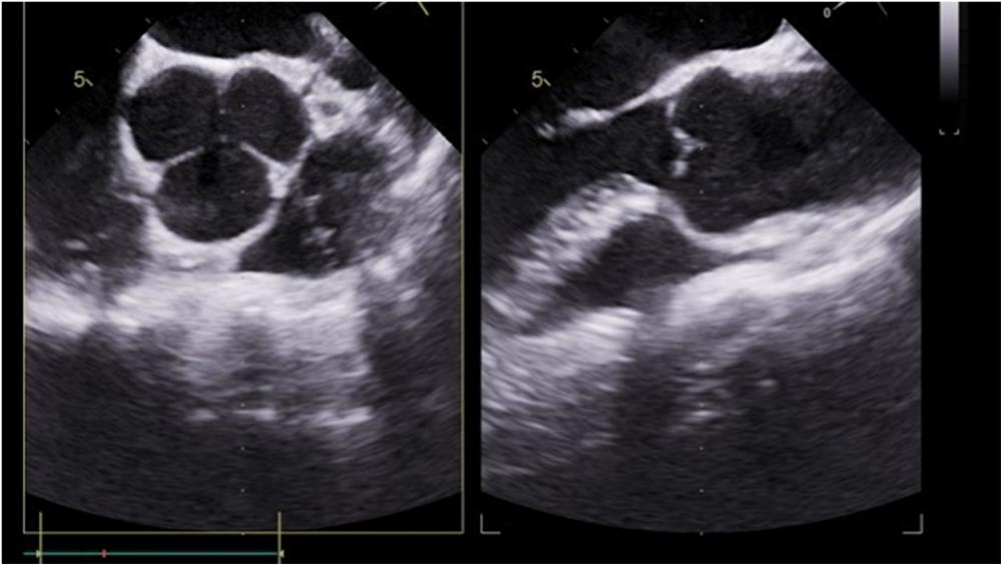
Transoesophageal echocardiography: normal looking aortic valve after antithrombotic treatment.

A consultation was obtained from the Heart Team on the 12th day of hospitalization, who decided to perform a follow-up TEE and surgical treatment if the mass was found again or progression was observed. No abnormalities on the aortic valve were detected in this TEE, indicating indirect evidence of thrombus (*Figure 5*). The patient was discharged in good condition and continued on the pharmacotherapy implemented in the hospital until a haematology consultation. One month later, a mild reduction in antithrombin III (AT III) activity (66%) was diagnosed without other causes of thrombophilia. This led to the replacement of enoxaparin with warfarin. An outpatient TEE was performed 2 months later and did not show any thrombi in the heart chambers despite questionable compliance with therapy (International Normalized Ratio < 2). Considering this, a decision was made to switch to rivaroxaban 20 mg daily.

## Discussion

Thrombosis of the native aortic valve, unlike thrombotic events on prosthetic valves, is a very rare disease; nevertheless, its reporting frequency has recently been increasing.^5^ Echocardiography has the highest sensitivity in detecting this pathology, but a transthoracic echocardiography may not be sensitive enough. Thrombophilia may cause co-occurring vascular complications in both the systemic and pulmonary systems, and its diagnosis is crucial for deciding the type of anticoagulation. The transient nature of native aortic valve thrombosis may be the reason for the underestimation of this clinical problem and inconsistent diagnoses. Considering this, a greater prevalence should be acknowledged beyond the statistical data based on case reports. Native aortic valve and aortic root thrombosis are clinically relevant, especially in patients presenting with embolic events. Because MI is the most common presentation, the current data highlight the need to keep a high index of suspicion, especially in patients with MI who have no culprit or have coronary thrombi without evidence of atherosclerosis.^5^

Each such patient requires rapid, multidirectional diagnostics, and their treatment poses a challenge for the treatment team. In this particular case, the detection of pulmonary embolism could have concluded the entire diagnostic and therapeutic process. Key factors included the elevated levels of D-dimers, which did not correlate with the area of pulmonary embolism, and the findings from echocardiography that led to the diagnosis of MI. The results from coronary angiography then directed the investigation towards secondary MI originating from valvular thrombosis, which was confirmed by TEE.

This scenario underscores the complexity of differential diagnosis in cardiology, where seemingly disparate findings can provide crucial insights into underlying pathologies. Elevated D-dimer levels are often associated with thromboembolic events, yet in this instance, they prompted a deeper investigation rather than confirming a singular diagnosis. Continuous advancements in imaging techniques and biomarkers are essential in refining diagnostic accuracy and treatment pathways, emphasizing the need for clinicians to remain vigilant and adaptable in their approach. It is worth noting that ECG-gated CT, performed as part of a triple rule-out (TRO) protocol during the initial diagnostic workflow in the emergency department, could have likely confirmed the correct diagnosis much earlier. The TRO CT scan allows for the simultaneous detection of pulmonary embolism, obstructive coronary disease, and aortic disease; however, it is not yet routinely available during emergency hospital services in our centre. In this particular case, the diagnosis of mildly reduced AT III activity does not support the hypothesis that this coagulopathy was the direct cause of aortic valve thrombosis. Antiphospholipid syndrome, protein C deficiency, and protein S deficiency are hypercoagulable states known to be associated with native aortic valve thrombosis.^5^ Although no such case has been reported regarding AT III deficiency in the medical literature to date, this finding in our patient should not be overlooked. The AT III deficiency is typically associated with venous thrombosis, while arterial thrombosis is rare. However, a few cases of multifocal coronary thrombosis associated with AT III deficiency have been reported.^6^ Some investigators suggest that venous thrombosis related to AT III deficiency may often mask arterial events, leading to an underestimation of their occurrence.^7,8^ Due to the lack of established recommendations, the decision regarding the choice and duration of antithrombotic pharmacotherapy in such cases undoubtedly presents a clinical challenge and requires an individualized approach.^9^ In our patient, considering the coronary intervention without stent implantation, a 6-month course of clopidogrel for antiplatelet therapy and indefinite rivaroxaban therapy were prescribed after assessing the bleeding risk.

## Data Availability

The data underlying this article will be shared on reasonable request to the corresponding author.

## Lead author biography

I have been a cardiology specialist for 15 years, working in a hospital and outpatient clinic in Poland. My main areas of professional interest are coronary artery disease and valvular disorders. In my daily practice, I perform coronary interventions as well as transthoracic and transoesophageal echocardiography.

**Consent:** The authors confirm that written consent for submission and publication of this case report has been obtained from the patient in line with the COPE guidelines.

**Funding:** None declared.
